# New Developments in the Germ Theory of Disease

**Published:** 1897-06

**Authors:** 


### New Developments in the Germ Theory of Disease.

It has been almost universally true in the history of science that a newly discovered fact or law looks astoundingly simple at first, but that with closer study so many exceptions and modifications of detail come to light that it is finally seen to be complex and intricate. So the heavenly bodies were first thought to move in circles, then closer observation elongated these into ellipses, and finally with more and more study a new crinkle was put into the orbit with every newly discovered perturbation, till now it is recognized that the actual curve described by a planet is exceedingly complex, tho of course its ground-plan is still an ellipse.

So, too, with the molecular theory of matter, the laws connecting pressure and temperature in gases, and so on. The latest fact to broaden out into such a complex system is the germ theory of disease. In a first statement it is delightfully simple. Every disease is due to a specific microbe. No microbe, no disease. Eliminate the microbe, cure the disease. But exceptions keep cropping out. Especially frequent are the cases in which the microbe is found, but not the disease, so that bacteriologists have come reluctantly to recognize that not the presence of the germ but some peculiar virulent condition of it causes the malady. In other words, the "diseased condition" is transferred from the patient to the germ, and it is now our business to find out what is the matter with the later. In an article discussing this interesting development the Paris correspondent of *The Medical News*, March 6, says:

"Probably the first thing that set the current of thought seriously in this direction was Roux and Yersin's discovery of the diphtheria bacillus in a large number of normal throats, and in parts of the country where diphtheria had not been epidemic for years. . . .

"After this came the description of various technical methods of differentiating two very important bacilli (Eberth's typhoid bacillus and the cholera bacillus) from a host of very similar bacilli rather widely spread in nature. For a good many years this effort at differentiation was thought to be successful, tho newer and constantly newer technical methods had to be invented, each more complicated than the last in order to enable the bacteriologist to decide with certainty as to the presence of the specifically pathogenic germ for these two diseases. . . .

"Now the leaders seem to be giving up the effort at further differentiation, and are coming around to the view that it is a family of bacilli one has to deal with in each of these diseases. Some of the members of each family are specially pathogenic and some are not. These may become virulent under certain conditions not well understood, but the main one seems to be the passage of the bacillus through one or more individuals whose lowered resistive vitality make them subject even to the originally attenuated virulence of the bacilli. This gain in virulence by passage through a series of animals is, of course, well known and founded on any number of observations."

The correspondent goes on to say that Dr. Roux, of antitoxin fame, has about come to the conclusion that these changes in virulence in microbes are the rule rather than the exception, and Metschnikoff, another great authority, found that the cholera bacillus "was widely spread in water in many places, practically all over the world, and that its specificity in the etiology of cholera was due to extrinsic circumstances that modified its virulence." The complexity that this introduces into all bacteriological tests is evident. Says the writer, in a final paragraph:

"Duclaux, who is Pasteur's successor as the Director of the Institute Pasteur, is giving a series of lessons on the biological chemistry of water. He, too, shares the opinion that it is a family of bacilli sometimes pathogenic, sometimes not, and widely spread in nature, that we are dealing with in the microbiology of typhoid and cholera. With regard to typhoid, he is as positive as is Metschnikoff regarding cholera. He referred especially to some work unpublished when he spoke, but which has since been reported to the Society of Biology. Two physicians in the military service at Val de Grace have found, as far as all bacteriological tests are concerned, true typhoid bacilli in the dust of the floors of wards in the hospital where there had been no typhoid cases for years. Duclaux deprecates that this seems to make doubtful the hygienic precautions with regard to water-supply that seemed so simple and so sure. For it would seem that these bacilli are so widespread in nature and so liable to become pathogenic, tho they may not have been before virulent, that the simple hygienic precaution of protecting the water-supply from human contamination, thought all-sufficient up to this time, may prove illusory."

A LADY writer points out the fact as worthy of note, that, while the men who commit suicide are almost always unmarried, the women who thus leave the planet are married. This leads her to the inference that, while men cannot live without women, many women find life unbearable with men.

